# Parameters associated with diagnosis of COVID‐19 in emergency department

**DOI:** 10.1002/iid3.440

**Published:** 2021-05-07

**Authors:** Claudio Ucciferri, Luca Caiazzo, Marta Di Nicola, Paola Borrelli, Michela Pontolillo, Antonio Auricchio, Jacopo Vecchiet, Katia Falasca

**Affiliations:** ^1^ Department of Medicine and Science of Aging, Clinic of Infectious Diseases University “G. d′Annunzio” of Chieti‐Pescara Chieti Italy; ^2^ Laboratory of Biostatistics, Department of Medical, Oral, and Biotechnological Sciences University “G. d′Annunzio” of Chieti‐Pescara Chieti Italy

**Keywords:** ED, heart failure, respiratory disease, SARS‐COV2, Sepsis disease

## Abstract

**Objectives:**

We designed this study to identify laboratory and radiological parameters, which could be useful to guide the clinician, in the evaluation of a suspected case of coronavirus disease 19 (COVID‐19).

**Methods:**

This retrospective, observational, single‐center‐study recruited patients with a suspect of COVID‐19 data were extracted from electronic medical records using a standardized data collection form.

**Results:**

A total of 566 patients with suspect COVID‐19 infection were enrolled (280 were COVID‐19+). The COVID‐19 population was characterized with bilateral‐pneumonia, a lower count of neutrophil, lymphocyte and monocyte, a lower neutrophil to lymphocyte‐ratio (NLR). Lower of platelet count, d‐dimer, troponin I, and serum calcium were in COVID‐19 patients. The occurrence of COVID‐19 diagnosis increased, independently of other variables, with pneumonia (odds ratio [OR]: 3.60; *p* < .001), neutrophil below normal range (OR: 4.15; *p* < .05), lactate dehydrogenase (OR: 2.09; *p* < .01) and sodium above normal range (OR: 2.34; *p* < .01). In patients with possible respiratory acute affections we found a higher neutrophil, higher monocyte, a higher NLR and a more elevation in d‐dimer. In the Sepsis group showed higher level of white blood cell, C‐reactive protein, d‐dimer, and procalcitonin.

**Conclusions:**

Our study confirms that patients with COVID‐19 have typical radiological and laboratory characteristics. The parameters highlighted in the study can help identify COVID‐19 patients, also highlighting which are the main differential diagnoses to be made and the parameters that facilitate the differential diagnosis.

**Abbreviations**
ALT: alanine aminotransferase
aPTT: activated partial thromboplastin time
AST: aspartate aminotransferase
AUC: area under the curve
CAP: community acquired pneumonia
COPD: chronic obstructive pulmonary disease
COVID‐19: coronavirus disease 19
CRP: C‐reactive protein
CT: computed tomography
CVD: cardiovascular diseases
ECDC: European Center for Disease Prevention and Control
ED: emergency department
eGFR: estimated glomerular filtration rate
HF: heart failure
LDH: lactate dehydrogenase
OD: odds ratio
ORF: Open Reading Frame
PT: prothrombin time
RD: respiratory diseases
RDT: rapid diagnostic test
RT‐PCR: reverse transcription polymerase chain reaction
SARS‐Cov‐2: severe acute respiratory syndrome coronavirus 2
WBC: white blood cell
WHO: World Health Organization

## 1 INTRODUCTION

Coronavirus disease 19 (COVID‐19) resulting from severe acute respiratory syndrome coronavirus 2 (SARS‐CoV‐2) infection, which is emerging as an unicum related to high infectivity and global diffusion, has became a pandemic. As the 30th of November 2020 the COVID‐19 pandemic is still raging all around the world with more than 63 million of cases and a tremendous deaths toll.^1^ Even if almost a year has passed since its start, the SARS‐CoV‐2 infections continue to be a public health threat for a lot of countries: in the United States and in India, where the infection has never really showed a decrease of cases, as well as in Europe, where after a partial slow down of contagions during the summer months now we observe a new increase in cases.^2^ The clinical spectrum of SARS‐CoV‐2 infection ranges from asymptomatic to severe cases presenting with refractory hypoxemia requiring invasive mechanical ventilation and death; this spectrum is related by a different therapies^3^, ^4^, ^5^, ^6^, ^7^ and with different outcome.^8^, ^9^, ^10^ Therefore, COVID‐19 condition required early and correct identification. Usually the emergency department (ED) is where a suspect COVID‐19 case is evaluated. During the first months of pandemia, ED faced a high number of suspects. Since during those months there were no rapid diagnostic tests (RDTs) for SARS‐CoV‐2, clinicians had only symptoms/signs, laboratory values and radiology parameters to identify a case of COVID‐19.^11^

However, at present, rapid tests are part of routine care and evaluation of COVID‐19 suspects. Dinnes et al.^12^ analyzed the performance of rapid tests, either molecular or antigen based, in a Cochrane review. They found an average sensitivity for antigen tests of 56.2% with an average specificity of 99.5%, while the sensitivity (95.2%) and specificity (98.9%) of molecular rapid tests were much better. Even so, the authors concluded stating that, with the studies currently available, they could not be certain of how these tests performed in clinical practice. RDTs could be useful to inform triage of reverse transcription polymerase chain reaction (RT‐PCR) lab based tests.^12^

RT‐PCR based SARS‐CoV‐2 tests are the standard for diagnosis, however, they suffer of variable sensitivity in response to a numbers of factor (like timing of testing relative to exposure, adequacy of specimen collection, specimen source),^13^ and they need time and an adequate laboratory to be processed.^14^ Therefore, they are of little help to diagnose a COVID‐19 in the emergency room.

The months to come will probably stress the ED, not only because of the surge of COVID‐19 cases but also for the seasonal trend of Upper respiratory infections and Influenza. Clinicians need to rely on valuable and rapid methods to identify suspect cases of COVID‐19. Laboratory examinations as well radiology exams are, with patient's symptoms and signs, available and fast tools to help the clinician through the diagnostic process in the ED.

With these backgrounds, we designed this study to identify laboratory and radiologic parameters, which could be useful to guide the clinician, in evaluating a suspect case of COVID‐19.

## 2 MATERIALS AND METHODS

### 2.1 Study design and population

This retrospective, observational, single‐center cohort study recruited all adults patients with a suspect of COVID‐19, admitted to the emergency room of the “SS. Annunziata” Clinical Hospital of Chieti, Italy, from 2 March 2020 to 25 May 2020.

The inclusion criteria were to be a suspect of COVID‐19 as defined by ECDC and World Health Organization (WHO) criteria.^15^

Epidemiological, demographic, clinical, laboratory findings and outcome data were extracted from electronic medical records using a standardized data collection form. All data were checked by two physicians (L.C. and M.P.), and a third researcher (P.B) adjusted any difference in interpretation between the two primary investigators.

Patients' data were subsequently divided in two categories, COVID‐19 and NO‐COVID‐19, on the basis of the results of SARS‐CoV‐2 nasoparhyngeal and oropharyngeal swab. We used RT‐PCR assay multiplex gene detecting Open Reading Frame‐1ab, N and S protein.

The NO COVID‐19 patients were then included in a pool of other diagnosis; the diagnosis of each patient has been defined from the discharge letter.

Final classification (COVID‐19 or NO COVID‐19 patient) were obtained from the discharge letters issued by the wards and from the results of the nasofaringeal carry out by RT PCR for SARS‐CoV‐2. All adult patients were diagnosed with COVID‐19 according to WHO interim guidance: they had clinical symptoms of COVID‐19 and confirmation of SARS‐CoV‐2 infection through instrumental signs and a positive result on RT‐PCR assays of nasopharyngeal swab specimens.

### 2.2 Data collecting

Selected data were encoded to create an anonymized dataset. Clarifications were discussed with the epidemiology department encoders or clinical teams. The analysis was undertaken after clinical outcomes were available for all patients.

Laboratory assessments consisted of a complete blood count, blood chemical analysis, coagulation testing, assessment of liver and renal function, and measures of electrolytes, C‐reactive protein (CRP), procalcitonin, lactate dehydrogenase (LDH), and creatine kinase. Lymphocytopenia was defined as a lymphocyte count of less than 1100 cells per cubic millimeter while thrombocytopenia as a platelet count of less than 150,000 per cubic millimeter.

We determined the presence of a radiologic abnormality based on the documentation or description in medical charts; if imaging scans were available, they were reviewed by attending physicians who extracted the data. If pneumonia was present, we defined the presentation pattern in: (1) ground glass; (2) consolidation; (3) mixed; (4) not definable (computed tomography not available, patient had only chest x‐ray).

The laboratory parameters were subsequently recoded into three levels (0 =* normal range*, 1 = *below normal range*, 2 = *above normal range*) using the normal range assessed by Clinical Pathology Laboratory of the “SS. Annunziata” Clinical Hospital of Chieti, for implementation of crude odds ratio analysis and multivariable logistic regression model.

Aims of the study

- We collected the results which primary endpoint to try to identify laboratory and radiological parameters that allow you to make a COVID‐19 diagnosis in emergency room and try to define the presence of predictive parameters for the diagnosis of COVID‐19;
- Secondary study endpoints were also assessed COVID Try to identify laboratory parameters predictive of prognosis in COVID‐19 populations;
- Compare laboratory and radiology characteristics of COVID patients and NO COVID patients, who were admitted in the ED with symptoms suggestive of COVID‐19, but in which the nasopharyngeal and oropharyngeal swab for SARS‐CoV‐2 were negative.

This study was performed in accordance with the principles embodied in the Declaration of Helsinki, and all participants provided written informed consent. This study was approved by the Ethics Committee at the University “G. d′Annunzio” Chieti‐Pescara and informed consent was waived due to the observational nature of the study.

Selected data were encoded to create an anonymized dataset. Clarifications were discussed with the epidemiology department encoders or clinical teams. The analysis was undertaken after clinical outcomes were available for all patients.

### 2.3 Statistical analysis

Descriptive analysis was carried out using median and interquartile range (IQR) for the quantitative variables and percentages values for the qualitative ones. Normality distribution for quantitative variables was assessed by the Shapiro–Wilk Test. The association between endpoint variable (COVID‐19) and explicative variables was investigated by Pearson *χ* ^2^ test and nonparametric Wilcoxon rank‐sum test for unpaired two‐samples or Kruskal–Wallis's test followed by the appropriate post hoc test if significant. The Bonferroni's correction for multiple comparisons tests was applied. Crude odds ratio (ORs) and corresponding 95% confidence interval (CI) were calculated to quantify the risk associated with the previously considered explicative variables for the endpoint variable using the Wald test. Multivariable logistic regression model was done to identify the mutually adjusted effect among COVID‐19/NO COVID‐19 diagnosis and the independent variables chosen on the basis of (1) the statistical significance (univariate analysis, *p* ≤ .05); (2) the clinical judgment and their contribution to the model fit (Likelihood‐Ratio test).^16^ The goodness of fit of the multivariable logistic regression model was assessed by the Hosmer–Lemeshow test and the area under the receiver operator characteristics (ROC) curve has been calculated to measure the accuracy of the model. We also performed internal validation of the model using k‐fold cross validation. The original sample is randomly partitioned into 10 equal sized subsamples for ROC curve comparison of the predictive model. The mean ROC for each k‐fold was then reported with 95% CIs

Statistical significance was set at the level of ≤0.05, unless adjustment for multiple comparisons (applying the Bonferroni correction) was needed. All analyses were performed using Stata software v15.1 (StataCorp).

## 3 RESULTS

A total of 556 patients with suspected COVID‐19 infection who were admitted to the ED of SS. Annunziata Clinical Hospital from 2 March 2020 to 25 May 2020, were included in this study. Of these, 280 (49.4%) were identified as laboratory‐confirmed COVID‐19.

Our population in general showed a slight predominance of male (53.5%) and had a median age of 72 (IQR: 55.0–84.0) years. Radiological signs of pneumonia were present in 202 patients (35.9%); pneumonia was bilateral in 76.7% of cases. Pleural effusion was noted in 146 subjects (25.9%). A total of 127 patients died overall in the cohort of study. The parameters of the study population are summarized in the Table 1.

**Table 1:** Baseline, laboratory, and radiological characteristics of study populations

	Total	no covid‐19	covid‐19	
	*N* = 566	*N* = 286	*N* = 280	*p* Value
Gender				
Male	303 (53.5%)	151 (52.8%)	152 (54.3%)	.723
Fale	263 (46.5%)	135 (47.2%)	128 (45.7%)	
Pneumonia				
Yes	202 (35.9%)	145 (50.7%)	220 (79.4%)	<.001
No	361 (64.1%)	141 (49.3%)	57 (20.6%)	
Pneumonia distribution				
Unilateral	84 (23.3%)	54 (38.0%)	30 (13.7%)	<.001
Bilateral	277 (76.7%)	88 (62.0%)	189 (86.3%)	
Pleural effusion				
No	417 (74.1%)	192 (67.1%)	225 (81.2%)	<.001
Yes	146 (25.9%)	94 (32.9%)	52 (18.8%)	
Outcome				
Survivor	429 (77.2%)	237 (82.9%)	192 (71.1%)	.001
Not survivor	127 (22.8%)	49 (17.1%)	78 (28.9%)	
Age (years)	72.0 (55.0–84.0)	72.0 (55.0–84.0)	72.0 (55.0–85.0)	.336
Neutrophil (cell/µl)	5890.0 (3710.0–9440.0)	7670.0 (4770.0–11740.0)	4485.0 (3130.0–6870.0)	<.001
Lymphocite (cell/µl)	1070.0 (720.0–1520.0)	1135.0 (680.0–1730.0)	1010.0 (740.0–1375.0)	.042
Monocyte (cell/µl)	530.0 (360.0–810.0)	660.0 (410.0–970.0)	460.0 (320.0–620.0)	<.001
N/L ratio	5.4 (3.0–10.7)	6.7 (3.6–13.8)	4.3 (2.5–8.0)	<.001
L/M ratio	2.1 (1.3–3.2)	1.8 (1.0–3.1)	2.3 (1.5–3.4)	<.001
crp (mg/L)	49.3 (13.3–133.9)	38.4 (6.9–145.7)	56.9 (22.3–128.6)	.047
d‐Dimer(mg/L)	1.1 (0.6–2.6)	1.3 (0.6–3.4)	0.9 (0.5–1.9)	.011
ldh (U/L)	240.0 (172.0–322.0)	214.0 (158.0–275.0)	265.0 (184.0–374.0)	<.001
Troponin I (pg/L)	14.2 (4.2–54.3)	17.9 (4.0–75.8)	12.2 (4.7–36.0)	.044
plt × 10 ^3^ (cell/µl)	210.0 (157.0–277.0)	227.0 (166.0–309.0)	196.0 (153.0–247.0)	<.001
pt (%)	91.6 (75.6–102.6)	86.7 (70.5–100.2)	95.1 (83.2–105.0)	<.001
ptt (sec)	32.0 (29.0–37.0)	32.0 (29.0–37.0)	32.0 (29.0–37.0)	.519
Creatinine(mg/dl)	1.0 (0.8–1.4)	1.0 (0.8–1.5)	0.9 (0.8–1.3)	.017
gfr (ml/min)	70.6 (40.3–97.5)	66.3 (34.8–99.8)	72.8 (45.1–94.1)	.400
ast (U/L)	24.0 (17.0–41.0)	22.0 (16.0–37.0)	28.0 (19.0–46.0)	<.001
alt (U/L)	21.0 (14.0–37.0)	20.0 (13.0–35.0)	24.0 (15.0–38.0)	.092
Procalcitonin (ng/ml)	0.1 (0.0–0.6)	0.2 (0.0–1.1)	0.1 (0.1–0.4)	.019
Sodium (mmol/L)	138.0 (135.0–140.0)	138.0 (136.0–141.0)	137.0 (135.0–140.0)	.024
Potassium (mmol/L)	4.1 (3.7–4.4)	4.1 (3.8–4.5)	4.0 (3.7–4.4)	.033
Calcium (mmol/L)	8.6 (8.2–9.2)	8.9 (8.3–9.3)	8.4 (8.0–8.9)	<.001

*Note: N* (%) or median and interquartile range (IQR) are shown when appropriate.

Abbreviations: ALT, alanine aminotransferase; AST, aspartate aminotransferase; COVID‐19, coronavirus disease 19; CRP, C‐reactive protein; LDH, lactate dehydrogenase; PT, prothrombin time; PTT, partial thromboplastin time.

A total of 280 subjects were diagnosticated with COVID‐19 while NO‐COVID‐19 cohort there were 286 subjects. The NO‐COVID‐19 cohort represented the control group in this study. The median age was the same in both group. We had a higher rate of death in the COVID‐19 group (28.9%) in comparison to the NO‐COVID‐19 cohort (17.1%).

Univariate analysis showed that COVID‐19 diagnosis was differently associated with all the demographic, laboratory and radiological characteristics except for age (*p* = .336), gender (*p* = .723), partial thromboplastin time (PTT) (*p* = .519), alanine aminotransferase (ALT) (*p* = .092) and estimated glomerular filtration rate (EGFR) (*p* = .400) (Table 1).

### 3.1 Variables associated with COVID‐19 diagnosis: crude OR and logistic regression analysis

**Figure 1:**
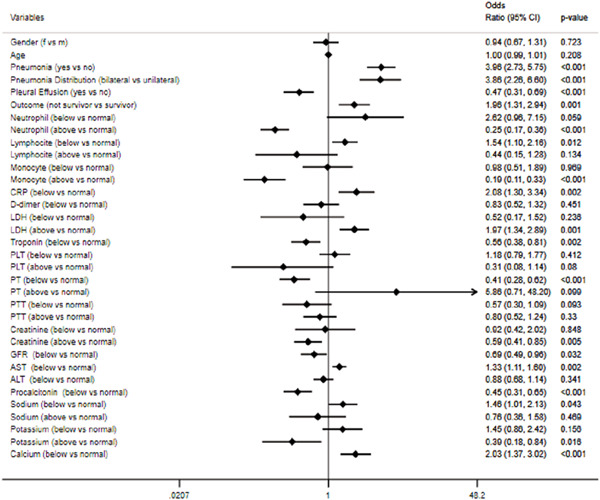
Crude OR and 95% Cl for identifying factors associated with COVID‐19 diognosis *The laboratory parameters were recoded into three levels: normal range (reference parameter), below normal range, and above normal range. Cl, confidence interval; COVID‐19, coronavirus disease 19; OR, odds ratio

Figure 1 reports crude OR calculated for all explicative variables on COVID‐19 diagnosis. In detail the strength of the association, measured by the value of OR concerns pneumonia, pneumonia bilateral distribution, negative outcome, above normal range values of CRP, LDH, aspartate aminotransferase (AST), hypocalcemia, and with below normal range values of lymphocytes and sodium. Adjusted OR refer to the logistic regression model shows that the occurrence of COVID‐19 diagnosis increased, independently of other variables, with pneumonia status (OR: 3.60; 95% CI: 2.20–5.90; *p* < .001) with neutrophil values below normal range (OR: 4.15; 95% CI: 1.03–16.74; *p* = .045) with LDH values above normal range (OR: 2.09; 95% CI: 1.28–3.41; *p* = .003) and with sodium values above normal range (OR: 2.34; 95% CI: 1.37–4.02, *p* = .002). Otherwise, the occurrence decreased with pleural effusion status (OR: 0.30; 95% CI: 0.17–0.53; *p* < .001) with neutrophil values above normal range (OR: 0.28; 95% CI: 0.17–0.47; *p* < .001) and with monocyte values above normal range (OR: 0.21; 95% CI: 0.09–0.46; *p* < .001).

**Figure 2:**
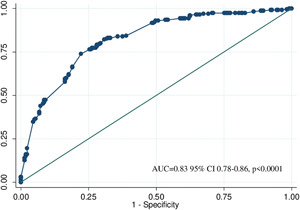
ROC Curve with AUC values (logistic regression model) for COVID‐19 diagnosis. AUC, area under the curve; COVID‐19, coronavirus disease 19; ROC, receiver operator characteristics

**Figure 3:**
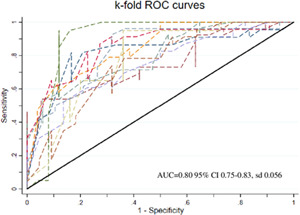
K‐fold (10) ROC Curve with mean AUC values. AUC, area under the curve; ROC, receiver operator characteristics

The Hosmer–Lemeshow indicated a good fit of the model in describing the data (*χ* ^2^ (451) = 91.55; *p* = .348). Figures 2 and 3 show the area under the curve (AUC) of predictive model (AUC = 0.83; 95% CI: 0.78–0.86; *p* < .0001) and the mean AUC for k 10 subsamples (AUC = 0.80; 95% CI: 0.75–0.83, *SD*: 0.056).

### 3.2 Description of COVID‐19 characteristics in our study

Focusing on the 280 COVID‐19 cohort, we found typical trends in some parameters. COVID‐19 patients showed in 69.6% of cases a normal range in neutrophil count, with a 7.5% cases of neutropenia and in 22.9% an elevation of neutrophil count.

Lymphopenia was a common tract shared by 58.9% of cases, as well as the elevated values of CRP (88.8%), the rise of d‐dimer (76.7%) and of LDH (64.5%). As recently described in literature, we found also a high (normal range: 0.52–3.78) neutrophil to lymphocyte ratio (NLR) in 60.4% of the patients.

The majority of our cohort had normal values of monocyte (85.7%), troponin I (66.2%), procalcitonin (68.2%), estimated glomerular filtration rate (eGFR) (62.3%), platelet (75.5%), prothrombin time (PT) (76.5%), PTT (72.3%), AST (59.8%), ALT (89%), sodium 62.7%) and potassium (81.7%). Interestingly, almost half of COVID‐19 patients showed lower levels of Calcium than the normal range (45.4%). The main radiological feature of these patients is a pneumonia (79.4%) with bilateral distribution (86.3%) with a prevalence of a ground‐glass pattern in 43.4% and a mixed pattern (Ground‐glass + consolidation) in 31.7% of cases. Pleural effusion was an uncommon finding, present in 18.8% of cases.

### 3.3 Comparison of parameters between COVID‐19 patients and other diagnoses

As explained previously, we divided the NO COVID‐19 patients into three diagnostic groups, as follows: respiratory disease (RD) (COPD, Upper respiratory infectious disease, community acquired pneumonia [CAP]), cardiovascular diseases (CVDs) (heart failure, Myocardial infarction, ischemic stroke, pulmonary embolism), and other infectious disease/sepsis group (abdominal infection/sepsis, urological infections/sepsis, soft tissue infections/sepsis). In NO COVID‐19 group, RDs represented the 37.4%, CVDs the 26.2%, and the other infectious disease/sepsis group constituted the 22%. We then compared each of the above group with COVID‐19 cohort.

### 3.4 COVID‐19 versus CVD

COVID‐19 patients were 280 while those diagnosed with CVD were 41. The proportion of male was similar, 54.3% in the COVID‐19 group and 53.7% in CVD group. Patients diagnosed with a CVD were older (median age 81 vs. 72). As just reported 78 COVID‐19 (28.9%) patients died in comparison with 8 deaths in the other group (19.5%). In CVD group, patients were on average 10 years older than in the COVID‐19 group. Pleural effusion was commoner in CVD than in COVID‐19. COVID‐19 subjects showed an higher neutrophil and monocyte count, as well an higher N/L ratio; on the other hand CRP was lower in CVD than in the COVID‐19 group. Troponin I was higher in CVD with a median value of 49.5 pg/ml, compared with a median 12.2 pg/ml in COVID‐19 patients. CVD patients had a reduced renal function with a mean eGFR of 45.6 mg/dl (45.6 vs. 72.8 mg/dl, *p* < .001). PT value was 95.1 in COVID‐19 and 75.9 in CVD patients. In this comparison, there was also a significant difference between the value of potassium, higher in CVD group than in COVID‐19. There was no statistical difference in LDH values between the two groups (Table 2).

**Table 2:** Differential diagnosis COVID‐19 versus other diagnosis (heart failure/CV disease, respiratory disease, Sepsis disease)

	COVID‐19	Heart failure/CV disease	Respiratory disease	Sepsis disease	
	*N* = 280	*N* = 41	*N* = 79	*N* = 60	*a*
Gender					
Male	152 (54.3%)	22 (53.7%)	41 (51.9%)	30 (50.0%)	.933
Female	128 (45.7%)	19 (46.3%)	38 (48.1%)	30 (50.0%)	
Age (years)	72.0 (55.0–85.0)	81.0 (76.0–87.0)*	75.0 (58.0–86.0)	79.0 (60.0–87.0)	.022
Outcome					
Survivor	192 (71.1%)	33 (80.5%)	61 (77.2%)	45 (75.0%)	.484
Not survivor	78 (28.9%)	8 (19.5%)	18 (22.8%)	15 (25.0%)	
Pneumonia distribution					
Unilateral	30 (13.7%)	6 (20.7%)	22 (37.9%)	8 (44.4%)	<.001
Bilatera	189 (86.3%)	23 (79.3%)	36 (62.1%)	10 (55.6%)	
Pleural effusion					
SI	225 (81.2%)	9 (22.0%)	58 (73.4%)	41 (68.3%)	<.001
NO	52 (18.8%)	32 (78.0%)	21 (26.6%)	19 (31.7%)	
Pleural effusion distribution					
Unilatera	25 (48.1%)	5 (15.6%)	13 (61.9%)	9 (47.4%)	<.001
Bilateral	27 (51.9%)	27 (84.4%)	8 (38.1%)	10 (52.6%)	
neutrophil (cell/µl)	4485.0	7720.0	6430.0	11080.0	<.001
	(3130.0–6870.0)	(6090.0–13680.0)*	(4020.0–9500.0)*	(7365.0–17445.0)*	
lymphocite (cell/µl)	1010.0 (740.0–1375.0)	1160.0 (720.0–1760.0)	1100.0 (760.0–1620.0)	1070.0 (595.0–1495.0)	.511
Monocyte (cell/µl)	460.0 (320.0–620.0)	770.0 (400.0–920.0)	570.0 (370.0–900.0)*	680.0 (465.0–1090.0)*	<.001
N/L ratio	4.3 (2.5–8.0)	7.3 (4.4–11.8)*	5.9 (3.3–10.5)*	12.8 (5.0–22.8)*	<.001
L/M ratio	2.3 (1.5–3.4)	1.8 (1.2–3.0)	1.9 (1.2–3.5)	1.3 (0.7–2.1)*	<.001
crp (mg/L)	56.9 (22.3–128.6)	23.3 (7.6–72.0)*	51.9 (12.6–149.3)	127.3 (41.6–229.8)*	<.001
D‐Dimer (mg/L)	0.9 (0.5–1.9)	1.2 (0.8–2.9)	1.4 (0.7–3.2)	1.6 (0.9–5.0)*	.001
ldh (U/L)	265.0 (184.0–374.0)	250.5 (181.0–341.0)	219.5 (167.0–280.5)*	214.0 (150.0–268.0)*	<.001
Troponin i (pg/L)	12.2 (4.7–36.0)	49.5 (19.3–131.4)*	17.3 (3.1–54.0)	18.5 (7.5–94.6)	<.001
plt × 10 ^3^ (cell/µl)	196.0 (153.0–247.0)	212.0 (170.0–285.0)	217.0 (158.0–319.0)	2225.0 (142.0‐310.0)	.091
pt (%)	95.1 (83.2–105.0)	75.9 (48.0–95.3)*	91.1 (75.1–103.4)	73.5 (64.1–87.8)*	<.001
ptt (sec)	32.0 (29.0–37.0)	32.0 (29.0–39.0)	32.0 (30.0–38.0)	35.0 (31.0–41.0)	.173
Creatinine (mg/dl)	0.9 (0.8–1.3)	1.3 (1.0–2.3)*	1.0 (0.8–1.5)	1.3 (0.8–2.5)*	<.001
gfr (ml/min)	72.8 (45.1–94.1)	45.6 (23.9–54.8)*	62.6 (38.1–92.3)	42.7 (21.3–91.0)*	<.001
ast (U/L)	28.0 (19.0–46.0)	26.0 (18.0–39.0)	22.0 (16.0–32.0)*	27.5 (16.0–50.0)	.009
alt (U/L)	24.0 (15.0–38.0)	28.0 (15.0–43.0)	18.0 (12.0–34.0)	23.0 (13.0–41.0)	.234
Procalcitonin (ng/ml)	0.1 (0.1–0.4)	0.2 (0.1–1.2)	0.2 (0.1–0.6)	1.6 (0.4–17.4)*	<.001
Sodium (mmol/l)	137.0 (135.0–140.0)	138.0 (133.5–139.5)	139.0 (135.0–141.0)	138.0 (135.0–142.5)	.224
Potassium (mmol/L)	4.0 (3.7–4.4)	4.5 (4.0–4.9)*	4.1 (3.8–4.4)	4.0 (3.6–4.5)	.001
Calcium (mmol/L)	8.4 (8.0–8.9)	8.6 (8.3–9.1)	8.7 (8.2–9.2)	8.7 (8.1–9.2)	.039

*Note: N* (%) or median and interquartile range (IQR) are shown when appropriate.

Abbreviations: ALT, alanine aminotransferase; AST, aspartate aminotransferase; COVID‐19, coronavirus disease 19; CRP, C‐reactive protein; LDH, lactate dehydrogenase; PT, prothrombin time; PTT, partial thromboplastin time.

* *p* Value less than α/3 for Bonferroni multiple testing correction other diagnoses versus COVID‐19.

### 3.5 COVID‐19 versus RD

In the NO‐COVID‐19 group, the respiratory affections were the most frequent diagnosis. The number of patients diagnosed within this group has been 79. Like in the COVID‐19 group, also in RD there was a predominance of the male gender (51.9%); 18 deaths occurred in the RD group (22.8%), a lower proportion than in the COVID‐19, although not significant. Median age was similar. RD had an higher neutrophil count as well as monocyte count had an higher NLR (5.9 vs. 4.3, *p* = .007). On the other side, LDH and AST were higher in COVID‐19 patients. The radiological presentations of RD were characterized by a unilateral pneumonia in 37.9% of the cases, a substantial higher proportion than COVID‐patients, in which unilateral abnormalities were present only in 13.7% of the cases (Table 2).

### 3.6 COVID‐19 versus Sepsis

In other infections/Sepsis group, there were patients diagnosed with abdominal sepsis, urological sepsis, skin and soft tissue infections/sepsis and other infections; this group were composed of 60 patients with an equal sex division (50% male and women) and a 25% of mortality (15 patients). In the Sepsis group, there was a significant higher level of almost all the WBC parameters, compared to COVID‐19: neutrophil count was 11,080 versus 4485 cell/µl, monocyte count was 680 versus 460 cell/µl, NLR was 12.8 versus 4.3. In contrast, lymphocyte to monocyte ratio was 1.3, lower than 2.3 of the COVID‐19 group. Moreover, Sepsis patients had higher CRP, d‐dimer, and procalcitonin values. Instead COVID‐19 had higher LDH values and a better renal function at the diagnosis. Even in this comparison, we noticed a higher PT value as well as lower calcium levels in COVID‐19 patients (Table 2).

## 4 DISCUSSION

The data showed that patients with COVID‐19 in emergency room have typical radiological and laboratory characteristics. These parameters can help identify COVID‐19 patients, also highlighting which are the main differential diagnoses to be made and the parameters that facilitate the differential diagnosis.

In COVID‐19 cohort, we found a lymphopenia in 58.9% of cases, elevated values of CRP, a rise of d‐dimer and of LDH. The neutrophil count was normal in 69.5%. Our observations are confirmed in the vast majority of review and meta‐analysis released until now.^13^, ^17^, ^18^, ^19^ In a systematic review by Fu et al.,^17^ involving 43 studies and 3600 patients, the most common abnormalities detected in COVID‐19 were decreased lymphocyte count (57.4%), an elevated CRP (68.6%) and an increase in LDH (51.6%). The authors found also a high incidence of bilateral pneumonia (73.2%) characterized by ground‐glass opacities (80%); as observed in our populations. Specifically, in our results the ground‐glass was present in 43.4% of cases, but probably this relative low percentage have to be attributed to our radiology pattern definition systems. In our categorization, in fact, we have created a specific category (mixed pattern) to include imaging that showed concomitant signs of ground‐glass opacities and consolidation. So, taking together ground‐glass pattern (43.3%) and mixed pattern (31.7%), we reach a percentage of 75%.

One of the common characteristics in COVID‐19 patients was also a high NLR. These finding is supported by the fact that a high NLR is a well‐known biomarker whose levels increase in wide‐spread inflammatory conditions.^20^ In COVID‐19, as we will discuss later, increasing levels of NLR appear to reflect disease severity.^21^, ^22^

An association, through univariate analysis, with COVID‐19 diagnosis has been reported for lymphopenia, CRP elevation, high levels of LDH and AST. On the contrary, procalcitonin above the normal range appears to reduce the probability of COVID‐19, as well as having only a consolidative pattern at the radiological examination. The association of higher values of procalcitonin, as well higher NLR, and an alternative diagnosis is related to the characteristics of our control group. In the comparison group (composed as mentioned by a large proportion of respiratory affections and sepsis), a high level of procalcitonin and NLR were common at the ED evaluation. Which is why the values of this two parameters are inversely associated to COVID‐19 diagnosis.

**Figure 4:**
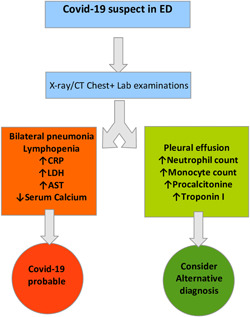
Parameters for COVID‐19 diagnosis. COVID‐19, coronavirus disease 19

A somehow predictive value has been shown also by low levels of calcemia, in accordance to the fact that almost half of our COVID‐19 patients had hypocalcemia at the admission (Figure 4).

As described before, common laboratory findings have been depicted for COVID‐19 in systematic reviews and meta‐analysis, but, to our knowledge, just a few have tried to detect potentially useful parameters in the early diagnosis of this disease.

Terpos et al.^23^ highlight in their critical review the dynamic of laboratoristic abnormalities in the early days of infection. In our cohort, we describe patients in their first 7–10 days of symptoms.

Assandri et al.^24^ compared COVID‐19 and NO COVID‐19 patients in the ED, describing how most COVID‐19 patients show a WBC count below 10 × 10^9^/L (82%), lymphocyte count below 1 × 10^9^/L (55.6%) as well as CRP, AST, and LDH elevation.^24^ Our results are corroborated also by Ferrari et al.^25^ in their work they compared laboratory findings of a mixed cohort of patients admitted to the ED of the San Raffaele hospital in Milan. They reported a statistical difference between COVID‐19 and NO COVID‐19 patients in the values of WBC, CRP, AST, ALT, and LDH.

Hypocalcemia observed in our COVID‐19 cohort, even if infrequently reported, has been recognized by Cappellini et al.,^26^ who described low levels of total and ionized calcium in COVID patients, compared to NO COVID, in the ED.

Our study showed an higher death rate in COVID‐19 than in the NO‐COVID‐19 group, this is interesting because compared with the rate of death of a group of alternatives diagnosis common in the ED.

During the first stage of the pandemia the EDs were overcrowded with patients with fever and/or cough and/or dyspnea, all suspected to be COVID‐19 affected.

However, now RDTs are available but, as we have seen before, they lack validation to pose a certain diagnosis of SARS‐CoV‐2.^23^ Furthermore, in case of a new upsurge of SARS‐CoV‐2 infections, Hospital and Health institutions will probably run out of stock.

Laboratory and radiology parameters are useful tools to guide the clinician in the evaluation of COVID‐19 patients.

At the best of our knowledge, only a few works have been published taking into account the potential differential diagnosis of COVID‐19 in the ED.

The existing studies investigated the difference between COVID‐19 patients and CAP or other respiratory infections.

In his work, Pan et al.^27^ reported a significant difference in nine laboratory features between COVID‐19 and patients with CAP. In a similar work Liang et al.^28^ observed that total WBC count and the neutrophil count were different between COVID‐19 and non‐COVID‐19 patients. Leukocytosis and neutrophilia were more common in patients with non‐COVID‐19 pneumonia.^28^

However, no one investigated until now the role of laboratory parameters to aid the differential diagnosis of Covid‐19 with other causes of fever, dyspnea, or cough.

In our results, we depicted potential useful laboratory features to guide the clinician to pose a differential diagnoses between COVID‐19 and three big groups of alternative diagnosis, particularly frequent in the ED settings, like respiratory affections, heart failure/CVDs and sepsis/bacterial infections.

When evaluating the patients with a possible respiratory acute affections, the RD are characterized by a higher neutrophil count, higher monocyte count, a higher NLR and a more pronounced elevation in d‐dimer. On the contrary COVID‐19 showed higher values of LDH and AST.

In patients with possible cardiovascular acute disease/heart failure the characteristic features are the presence of pleural effusion (usually bilateral), a higher neutrophil count, a higher monocyte count, a higher NLR, higher troponin I, a higher level of serum potassium. On the other hand, COVID‐patients have higher value of CRP, a more enhanced prolongation of PT. HF/CV patients show at the admission a worse renal function, with higher value of creatinine and decreased GFR.

Finally, patients with a Sepsis or a bacterial infection are characterized by higher neutrophil count, higher monocyte count, higher NLR, higher Lymphocyte to monocyte ratio, higher CRP, higher d‐dimer, higher troponin I, higher procalcitonin. Moreover Sepsis patients had a worse renal function than COVID‐19, with higher creatinine and lower GFR. Interestingly COVID‐patients showed a more prolonged PT and higher level of LDH.

These last two laboratory features assume an important role because no matter what the alternative diagnosis is or how severe is the clinical condition (like Sepsis): COVID‐19 display a higher LDH and a pronounced PT prolongation.

Our study has some major limitations. First of all, we could not include the comorbidities of each patients because they were not registered in the electronical medical systems we use to collect baseline characteristics, laboratory, and radiology features of recruited patients.

As explained before, to calculate the OR of mortality in COVID‐19 cohort, we use as control group No COVID‐19 patients with other acute diseases. This has probably affected our potency for some parameters.

Finally, our cohort of COVID‐19 patients was composed of 280 people, but the laboratory features were not available for all of them.

## 5 CONCLUSIONS

Our study confirm and outlined that COVID‐19 patients have common radiological and laboratory characteristics. Our results describe, as reported before in literature, that these common alterations of lab values are already present in the early phase of disease.

Nowadays incidence of SARS‐CoV‐2 infection is increasing day by day all over Europe. The ED are just now and probably will front an high burden of suspect COVID‐19 cases when the seasonal influenza will start to spread.

We demonstrated that some parameters are associated with COVID‐19, even in the first days of illness. We also showed that some common diagnosis in ED can be differentiated from COVID‐19 looking to a few laboratory values.

We believe that our results could be useful to help clinician to identify COVID‐19 in the emergency setting.

To conclude, the evaluation of laboratory exams and radiological characteristics are still a fundamental tool to identify COVID‐19 cases, even more in the early phases of illness during the evaluation of the suspect in ED.

## CONFLICT OF INTERESTS

The authors declare that there are no conflict of interests.

## AUTHOR CONTRIBUTIONS

Claudio Ucciferri and Luca Caiazzo were responsible for the conception, design, and write of this work. Marta Di Nicola and Paola Borrelli performed analysis and interpretation of all the data. Michela Pontolillo and Antonio Auricchio performed the data collection. Jacopo Vecchiet and Katia Falasca were revisioned the manuscript. All authors read and approved the final manuscript.

## Data Availability

The datasets used and/or analyzed during the current study are available from the corresponding author on reasonable request.
